# Gene Expression Changes and Associated Pathways Involved in the Progression of Prostate Cancer Advanced Stages

**DOI:** 10.3389/fgene.2020.613162

**Published:** 2021-01-21

**Authors:** Elena A. Pudova, George S. Krasnov, Anastasiya A. Kobelyatskaya, Maria V. Savvateeva, Maria S. Fedorova, Vladislav S. Pavlov, Kirill M. Nyushko, Andrey D. Kaprin, Boris Y. Alekseev, Dmitry Y. Trofimov, Gennady T. Sukhikh, Anastasiya V. Snezhkina, Anna V. Kudryavtseva

**Affiliations:** ^1^Engelhardt Institute of Molecular Biology, Russian Academy of Sciences, Moscow, Russia; ^2^National Medical Research Radiological Center, Ministry of Health of the Russian Federation, Moscow, Russia; ^3^National Medical Research Center for Obstetrics, Gynecology and Perinatology named after Academician V.I. Kulakov, Ministry of Health of the Russian Federation, Moscow, Russia

**Keywords:** prostate cancer, gene expression, PFS, TCGA, PRAD, WCDT-MCRPC

## Abstract

Prostate cancer (PC) is one of the most common cancers among men worldwide, and advanced PCs, such as locally advanced PC (LAPC) and castration-resistant PC (CRPC), present the greatest challenges in clinical management. Current indicators have limited capacity to predict the disease course; therefore, better prognostic markers are greatly needed. In this study, we performed a bioinformatic analysis of The Cancer Genome Atlas (TCGA) datasets, including RNA-Seq data from the prostate adenocarcinoma (PRAD; *n* = 55) and West Coast Dream Team – metastatic CRPC (WCDT-MCRPC; *n* = 84) projects, to evaluate the transcriptome changes associated with progression-free survival (PFS) for LAPC and CRPC, respectively. We identified the genes whose expression was positively/negatively correlated with PFS. In LAPC, the genes with the most significant negative correlations were *ZC2HC1A*, *SQLE*, and *KIF11*, and the genes with the most significant positive correlations were *SOD3*, *LRRC26*, *MIR22HG*, *MEG3*, and *MIR29B2CHG*. In CRPC, the most significant positive correlations were found for *BET1*, *CTAGE5*, *IFNGR1*, and *GIMAP6*, and the most significant negative correlations were found for *CLPB*, *PRPF19*, *ZNF610*, *MPST*, and *LINC02001*. In addition, we performed a gene network interaction analysis using STRINGdb, which revealed a significant relationship between genes predominantly involved in the cell cycle and characterized by upregulated expression in early recurrence. Based on the results, we propose several genes that can be used as potential prognostic markers.

## Introduction

Prostate cancer (PC) is the second most common cancer in men worldwide (Rawla, 2019). Advanced PC presents as locally advanced and castration-resistant tumors. Locally advanced PC (LAPC) is characterized by the spread of the tumor beyond the prostate capsule and is more aggressive than localized PC. Androgen deprivation therapy aimed at reducing the level of circulating testosterone is often used in LAPC treatment (Yap et al., 2016). However, despite rapid patient responses to this therapy, after 18–36 months, the disease frequently progresses to castration-resistant PC (CRPC; Yap et al., 2016). Metastatic CRPC is a prognostically unfavorable disease that requires regular systematic examination and monitoring and significantly impairs quality of life.

During therapy, in some patients, PC has an aggressive course, leading to metastasis, while in others, the disease has an indolent course, with a low tendency for progression. For prognostic assessment of the disease course, various clinical parameters are used, such as the level of prostate-specific antigen (PSA) and/or Gleason score; however, these parameters are not sufficiently informative (Keyes et al., 2013). To solve this problem, new molecular markers are needed that are highly associated with disease progression, which, when used in combination with existing clinical parameters, have a high predictive value. Currently, a promising area in the search for potential markers is the analysis of the most significant and consistent changes in the tumor transcriptome (Larkin et al., 2012; Cao et al., 2019; Pudova et al., 2019; Song et al., 2019).

An important criterion in the study of cancer data is progression-free survival (PFS). This parameter is the time from a random assignment in a clinical trial to disease progression or death from any cause (Forsythe et al., 2018). The study of PFS-specific molecular events will help to identify the major changes associated with the onset of disease progression.

This study aimed to analyze the transcriptome profiles of LAPC and CRPC based on the RNA-Seq data from the prostate adenocarcinoma (PRAD) and West Coast Dream Team – metastatic CRPC (WCDT-MCRPC) projects in The Cancer Genome Atlas (TCGA), respectively. We analyzed the changes in gene expression and molecular pathways related to PFS. The results of this study improve our understanding of the mechanisms underlying PC progression and identify molecules with potential as prognostic markers.

## Materials and Methods

### Data Collection

The study included RNA-Seq data for LAPC samples from the PRAD project (*n* = 55) and CRPC samples from the WCDT-MCRPC project (*n* = 84), which were donated to the TCGA consortium. We focused on the study of Caucasian patients (Caucasians were identified as “white” in the databases). The trimmed mean of M-values (TMM) method was used to normalize the RNA-Seq data of each dataset. Ethical approval was not available for the study as our data were revealed from the public database.

### Relative Gene Expression and Downstream Analysis

RNA-Seq data from the TCGA project (read counts per gene evaluated by HTSeq) were downloaded from the repository of GDC Data Portal^1^ and then analyzed in the statistical environment R.^2^ Data normalization and gene expression analysis with generalized linear models was performed using edgeR package (Robinson et al., 2010). The obtained results were considered statistical significance when the *p*-value of the quasi-likelihood F-test (QLF test) of <0.05. We did not use Benjamini-Hochberg (BH) *p*-value adjustment here because very few genes pass the threshold after it is applied.

In order to identify the genes whose expression change is most strongly associated with PFS, we evaluated Spearman’s rank correlation coefficient between the normalized gene expression level and PFS. Here, the obtained results were also considered statistically significant when both Spearman’s *p* < 0.05 and QLF test *p* < 0.05. For this analysis, we pre-filtered genes by the following parameters: read counts per million (CPM) > 8 and gene expression fold change is two- or (LogFC) > 1.

Next, we performed KEGG (Kyoto Encyclopedia of Genes and Genomes database) pathway enrichment analysis using the clusterProfile package (Yu et al., 2012). Additionally, we performed interaction network reconstruction and Gene Ontology (GO) pathway enrichment analysis using the STRINGdb (Szklarczyk et al., 2017). For enrichment analyses, BH adjustment to calculate the false discovery rate (FDR) was applied. The obtained results of this analysis were considered statistically significant when the FDR-value of <0.05. When constructing the networks, only data on direct protein-protein interactions were used (other associations such as co-expression, co-occurrence, gene fusions, and neighborhood, which are set by default, were excluded).

## Results

Using PRAD and WCDT-MCRPC datasets, we analyzed changes of the relative gene expression associated with PFS (the number of days to recurrence) and found 889 genes for the LAPC and 1,889 genes for CRPC with the *p* < 0.05 according to the QLF test (Supplementary Tables S1 and S2).

### Pathways Enrichment Analysis

**Figure 1:**
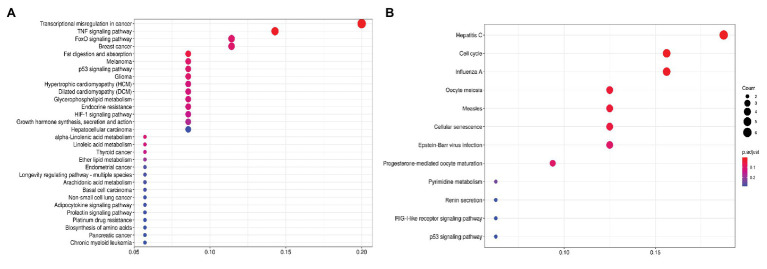
Dotplot showing the results of KEGG pathways enrichment analyses performed for top-80 upregulated **(A)** and downregulated **(B)** genes associated with PFS in LAPC. The *x*-axis indicates *k/n* ratio (“gene ratio”), where *k* is the number of genes participating in the current KEGG pathway in the top-80 list and *n* is the total number of genes that participate this pathway. Dot color indicates the false discovery rate (FDR) values according to the Fisher’s exact test.

First, we focused on the pathways enrichment analysis (KEGG database) to identify the major pathways, the expression of whose members may be positively or negatively associated with PFS (Figure 1). For this analysis, we selected top-80 genes, increased expression of which was positively associated with PFS (further – “upregulated genes”) and top-80 genes with negatively associated expression (further – “downregulated genes”). In LAPC, the most statistically significant enrichment with upregulated genes was noted for “Transcriptional misregulation in cancer” pathway (hsa05202). The analysis of downregulated genes showed a significant enrichment for the “Cell cycle” pathway (hsa04110; Table 1).

**Table 1:** Kyoto Encyclopedia of Genes and Genomes (KEGG) pathways that are significantly enriched with genes associated with progression-free survival (PFS) in locally advanced prostate cancer (LAPC).

KEGG ID	Pathway name	Enrichment *p*-value	Enrichment FDR	Genes
**Enriched with upregulated genes**				
hsa05202	Transcriptional misregulation in cancer	1,53E-05	1,98E-03	*CEBPB*, *RARA*, *GADD45B*, *CDKN1A*, *NR4A3*, *IGF1*, *SUPT3H*
hsa04668	TNF signaling pathway	1,12E-04	7,29E-03	*BCL3*, *CEBPB*, *JUNB*, *SOCS3*, *IRF1*
hsa04975	Fat digestion and absorption	8,31E-04	3,60E-02	*PLA2G5*, *CD36*, *PLA2G2A*
**Enriched with downregulated genes**				
hsa05160	Hepatitis C	2,81E-05	2,84E-03	*RSAD2*, *IFIT1*, *MX1*, *DDX58*, *OAS3*, *EIF2AK2*
hsa04110	Cell cycle	1,17E-04	5,89E-03	*E2F5*, *CCNB1*, *RAD21*, *CCNB2*, *BUB1*
hsa05164	Influenza A	5,08E-04	1,71E-02	*RSAD2*, *MX1*, *DDX58*, *OAS3*, *EIF2AK2*
hsa04114	Oocyte meiosis	1,57E-03	3,96E-02	*CALML3*, *CCNB1*, *CCNB2*, *BUB1*
hsa05162	Measles	2,07E-03	4,17E-02	*MX1*, *DDX58*, *OAS3*, *EIF2AK2*

**Figure 2:**
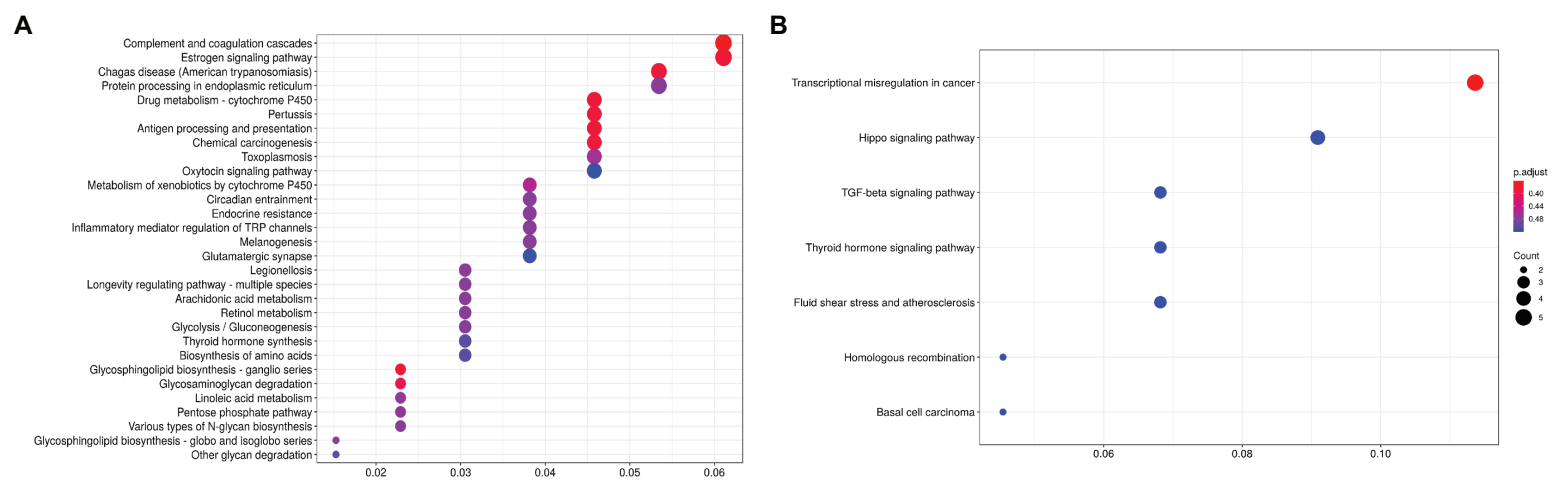
Dotplot showing the results of KEGG pathways enrichment analyses performed for top-250 upregulated **(A)** and downregulated **(B)** genes associated with PFS in CRPC. The *x*-axis indicates *k/n* ratio (“gene ratio”), where *k* is the number of genes participating in the current KEGG pathway in the top-250 list and *n* is the total number of genes that participate this pathway. Dot color indicates the FDR values according to the Fisher’s exact test.

For CRPC, a pathway enrichment analysis (top-250 up- and downregulated genes) revealed two pathways, “Complement and coagulation cascades” (hsa04610) and “Drug metabolism-cytochrome P-450” (hsa00982), which were enriched with genes that have expression positively associated with PFS. When considering pathways negatively associated with PFS, we have seen an overrepresentation of genes participating in such cancer-associated pathways as the “TGF-beta signaling pathway” (hsa04350), “Hippo signaling pathway” (hsa04390), and others (Figure 2; Table 2).

**Table 2:** KEGG pathways that are significantly enriched with genes associated with PFS in castration-resistant prostate cancer (CRPC).

KEGG ID	Pathway name	Enrichment *p*-value	Enrichment FDR	Genes
**Enriched with upregulated genes**				
hsa04610	Complement and coagulation cascades	8,14E-05	1,88E-02	*CPB2*, *SERPIND1*, *CFI*, *VTN*, *KNG1*, *SERPING1*, *SERPINA1*, *C3*
hsa00982	Drug metabolism – cytochrome P450	1,45E-04	1,88E-02	*ADH6*, *ADH1B*, *CYP3A4*, *CYP2E1*, *CYP2C8*, *GSTP1*
**Enriched with downregulated genes**				
hsa05202	Transcriptional misregulation in cancer	3,28E-03	3,65E-01	*HIST1H3C*, *TAF15*, *ASPSCR1*, *NCOR1*, *EYA1*
hsa04390	Hippo signaling pathway	1,04E-02	5,17E-01	*BMP4*, *TGFB2*, *BMP5*, *NKD1*
hsa04350	TGF-beta signaling pathway	1,45E-02	5,17E-01	*BMP4*, *TGFB2*, *BMP5*

### Correlation Analysis Between Gene Expression and PFS

Further, a correlation analysis between the gene expression and PFS was performed in LAPC and CRPC using Spearman’s rank correlation calculation.

**Figure 3:**
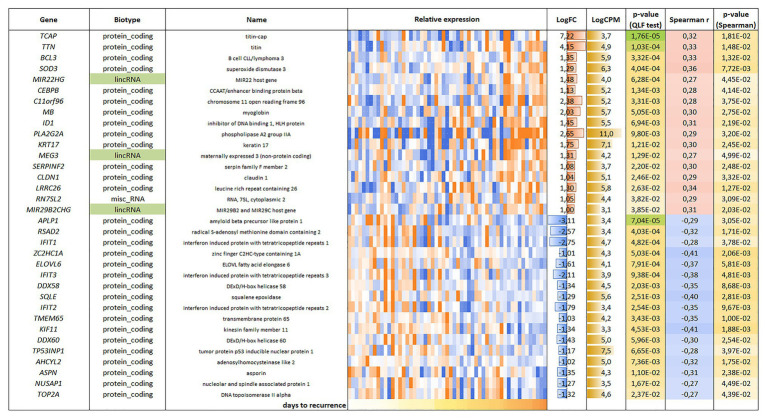
List and heatmap demonstrating log relative expression level of genes with significant Spearman’s rank correlation coefficient relative to PFS for LAPC. Cell colors (blue-white-orange gradient) correspond to the binary logarithm of the ratio of the expression level in a current sample to the average level across all the samples (per each gene). Blue – expression level is below the average, orange – above the average. LogFC – binary logarithm of the ratio of GLM-approximated expression values between samples with maximal and minimal PFS; LogCPM – binary logarithm of read counts per million (CPM); *p*-value (QLF test) – *p*-value according to quasi-likelihood F-test (edgeR); Spearman *r* – Spearman’s rank correlation coefficient between gene expression level and PFS; *p*-value (Spearman) – *p*-value according to Spearman’s rank correlation test.

For the LAPC, 34 genes were found to be correlated with PFS (*p* < 0.05); an equal number of genes had positive (17/34) and negative (17/34) correlation with PFS (Figure 3).

**Figure 4:**
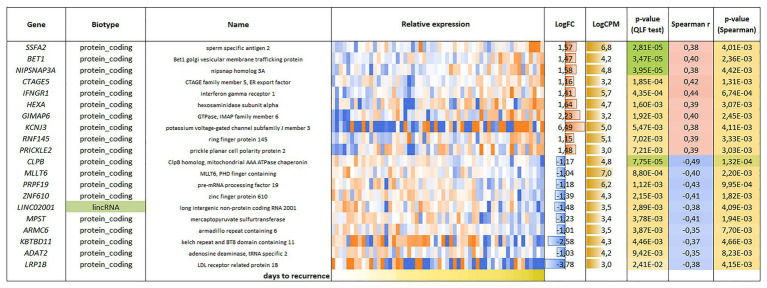
List and heatmap demonstrating log relative expression level of genes with significant Spearman’s rank correlation coefficient relative to PFS for CRPC. For comments, see the Figure 3 legend.

For the CRPC, 118 genes were found, including 81 genes with positive correlation and 37 genes with negative correlation with PFS (Figure 4).

#### Interaction Network Analysis Using STRINGdb

**Figure 5:**
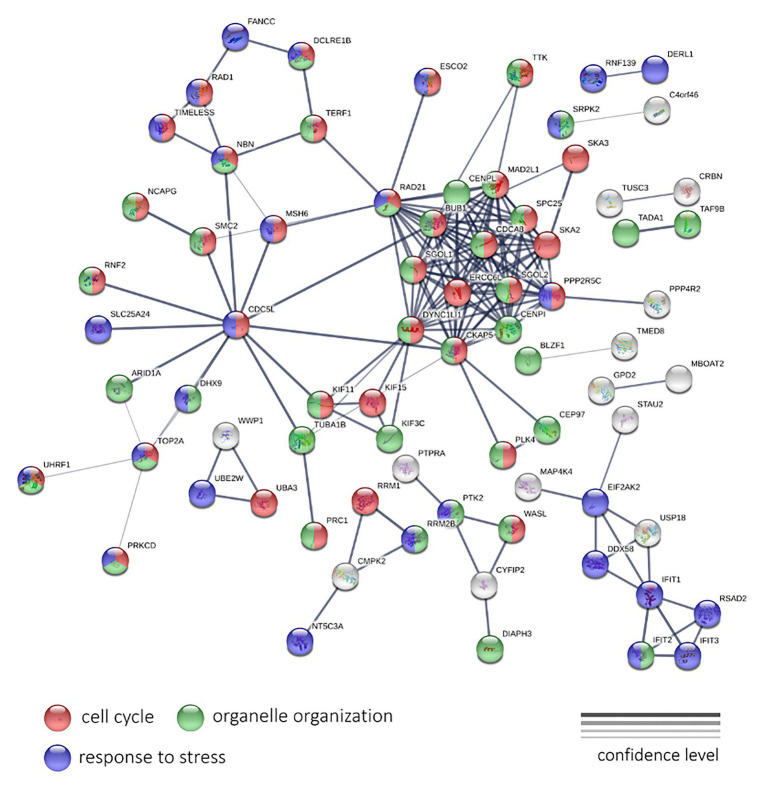
The protein-protein interaction network of 190 genes with a significant negative expression correlation with PFS in LAPC. Circle colors indicate proteins participating in the cell cycle regulation (red), organelle organization (green), and response to stress (dark blue). If a gene does not belong to these categories, it is marked with gray. The thickness of lines indicates the reliability of evidence of the interaction between two proteins (direct or indirect experimental confirmation, predictions from homologues, etc.).

To analyze the LAPC, we examined 190 genes, expression of which is negatively, and 115 genes – positively associated with PFS. These genes passed a threshold of *p* < 0.05 (both QLF test and Spearman’s rank correlation analysis), irrespectively of CPM and abs(LogFC) values. A statistically significant result was obtained for genes with a negative correlation. For these genes, the network has a strong enrichment of protein-protein interactions (PPI) with *p* < 1.0e-16 (Figure 5).

The genes shown in Figure 5 encode for proteins participating in such processes as the “Cell cycle” (GO: 0007049; enrichment FDR = 2.24e-15), “Organelle organization” (GO: 0006996; FDR = 8.53e-06), “Response to stress” (GO: 0006950; FDR = 0.0490) terms. We have also noted the cluster with the highest number of interactions, including *RAD21*, *SGOL1*, *DYNC1LI1*, *CKAP5*, *CENPI*, *PPP2R5C*, *SKA2*, *SPC25*, *MAD2L1*, *CENPL*, *BUB1*, *ERCC6L*, *SGOL2*, and *CDCA8* genes. Most of them participate cell cycle process.

**Figure 6:**
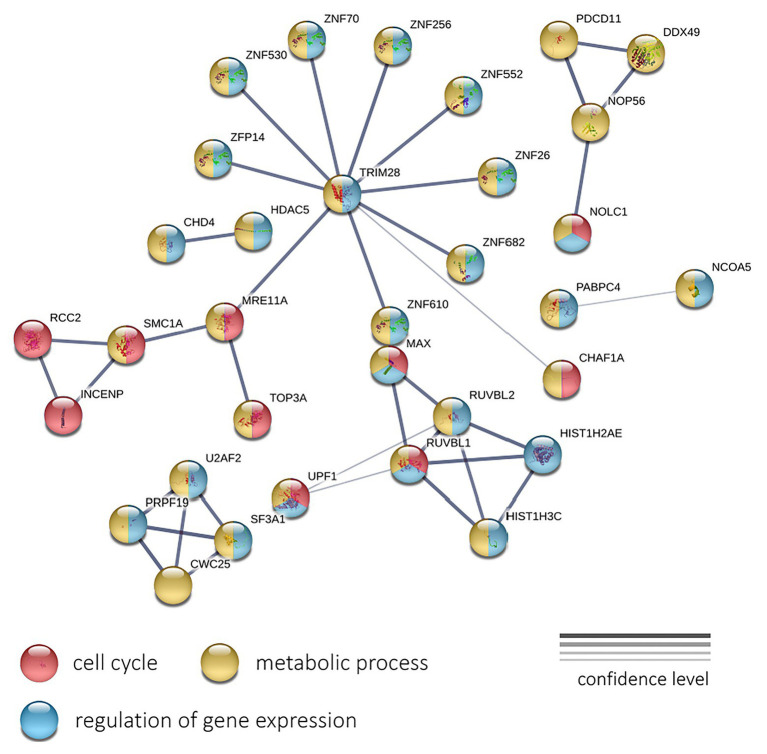
The protein-protein interaction network of 196 genes with a significant negative expression correlation with PFS in CRPC. Circle colors indicate proteins participating in the cell cycle regulation (red), metabolic process (yellow), and regulation of gene expression (light blue). The thickness of lines indicates the reliability of evidence of the interaction between two proteins (direct or indirect experimental confirmation, predictions from homologues, etc.).

Next, we performed a protein-protein interaction network analysis using 196 genes with negative expression correlation with PFS for CRPC. The analysis (PPI enrichment *p* = 0.000112) revealed involved enrichment in genes participating the “Cell cycle” (GO: 0007049; enrichment FDR = 0.0207), “Regulation of gene expression” (GO: 0010468; FDR = 0.0215), and “Metabolic process” (GO: 0008152; FDR = 0.0345) terms (Figure 6). The interaction network formed with genes positively correlated with PFS did not pass the PPI enrichment *p*-value threshold.

## Discussion

In the present study, we examined RNA-Seq data from the two most advanced stages of PC, LAPC and CRPC, with an aim to reveal the major changes in the transcriptome associated with disease progression within each analyzed category. The pathological manifestation of LAPC is invasion of the prostatic capsule as well as invasion beyond it, whereas CRPC, which is the next progressive stage, is characterized by the presence of distant metastases.

Using KEGG pathway enrichment analysis, we found several pathways, activation of which is possibly associated with PFS period, either positively or negatively. In LAPC, the most prominent associations were the increased expression of the participants of “TNF signaling pathway” and “Transcriptional misregulation in cancer.” Transcriptional misregulation involves an extensive network of processes that play dual roles in cancers, and probably in LAPC.

In CRPC, we identified putative activation of “Complement and coagulation cascades” and “Drug metabolism-cytochrome P-450” KEGG pathways to be associated with increased PFS time. According to various studies, the expression levels of complement system genes vary in different cancers. In PC, upregulated expression of complement system genes in both the classical and alternative pathways is associated with a good prognosis and long-term patient survival (Roumenina et al., 2019). Based on our results, putative activation of the complement pathway increased the relapse time in CRPC. Therefore, in patients with activation of the complement pathway genes, a longer response to therapy is expected, which may lead to the elimination of tumor-induced immunosuppression and increased antitumor immunity. Among the identified pathways enriched with genes negatively associated with PFS in CRPC, we noticed several cancer-associated pathways such as the “Hippo signaling pathway” and “TGF-beta signaling pathway” as well as “Transcriptional misregulation in cancer.”

Next, we performed PPI network and GO enrichment analysis using the STRINGdb. We examined the interaction networks for sets of genes that were positively and negatively correlated with PFS. In both datasets, statistically significant results were obtained only for genes with negative correlations. In LAPC, an extensive network of protein-protein interactions was identified for downregulated genes, which suggests close biological relationships among the genes and proteins under study. We observed significant enrichment with participants of several biological processes such as “Cell cycle,” “Organelle organization,” and “Response to stress.” In CRPC, we observed strong enrichment with genes involved “Cell cycle,” “Regulation of gene expression,” and “Metabolic process.” This suggests that the activation of the cell cycle and an increase in the expression of cell cycle-related genes may be behind the formation of more aggressive tumor phenotype and shortened PFS period, for both LAPC and CRPC. This is to be expected. Needless to say once again, the cell cycle, in particular cellular mitosis, and cancer are closely related, as cancer cells undergo abnormal, uncontrolled mitosis, which supports tumor growth and metastasis, two processes that are integral to disease progression (Weaver and Cleveland, 2005).

In our study, we also focused on identifying potential prognostic markers among the genes that have the strongest correlations with PFS. In LAPC, *KIF11* (Spearman’s rank correlation coefficient, *r_s_* = 0.41), *ZC2HC1A* (*r_s_* = −0.41), and *SQLE* (*r_s_* = −0.40) had the greatest negative correlations with PFS, while *SOD3* (*r_s_* = 0.36) and *LRRC26* (*r_s_* = 0.34) had the highest positive correlations. We reviewed the literature regarding the role of these genes in cancer, especially PC.

The kinesin family member 11 (*KIF11*) gene encodes mitotic kinesin, which plays a central role in mitosis (Wojcik et al., 2013). In PC, assessment of tumor cell differentiation, which is recorded as the Gleason score, is an important prognostic parameter. A value of 8 or higher corresponds to a poorly differentiated tumor and is associated with an unfavorable prognosis (Gordetsky and Epstein, 2016). A value of 8 or higher corresponds to a poorly differentiated tumor and is associated with an unfavorable prognosis. It was shown that *KIF11* gene expression was higher in PC tumor samples with a Gleason score of 8 (poorly differentiated tumors) than in tumor samples with a Gleason score of 7 (moderately differentiated tumors; Piao et al., 2017). In our study, we found that *KIF11* gene expression was negatively correlated with an increase in the number of days before recurrence, which confirms the relationship of this gene with unfavorable prognosis in PC.

There are few data regarding zinc finger C2HC-type containing 1A (*ZC2HC1A*). However, Zhu et al. (2019) showed that overexpression of the *ZC2HC1A* gene was associated with unfavorable prognosis in hepatocellular carcinoma. In our study, *ZC2HC1A* expression was increased in PC samples with early onset recurrence.

The *SQLE* gene encodes squalene epoxidase, which is involved in cholesterol synthesis. Cholesterol is an essential component of cell membranes and a precursor for the synthesis of androgens (Pelton et al., 2012). According to previous data, the *SQLE* gene is expressed in aggressive PC, and its expression is correlated with the Gleason score. It has been suggested that the progression of PC depends on the *de novo* synthesis of cholesterol catalyzed by *SQLE* (Stopsack et al., 2017). In our study, we observed higher *SQLE* gene expression in samples with early onset relapse.

Prostate cancer can also progress due to oxidative stress, which produces reactive oxygen species (ROS; Shiota et al., 2011). Cells are protected against ROS by antioxidant enzymes, such as superoxide dismutase (SOD), which functions as a first line antioxidant enzyme. The *SOD3* gene encodes an extracellular isoform of the enzyme, and data suggest that *SOD3* acts as a tumor suppressor in PC (Faraci and Didion, 2004). Thus, the effect of *SOD3* gene expression on cell proliferation, migration, and invasion of PC-3 cells was assessed, and the results showed that overexpression of *SOD3* inhibits these processes (Kim et al., 2014). Our data also demonstrate that *SOD3* gene expression was increased relative to time to relapse and was lower in tumor samples from patients with an unfavorable prognosis.

Studies on the function of *LRRC26* gene in LNCaP cell culture have shown that its overexpression leads to suppression of the NF-κB pathway, which is involved in cancer progression and metastasis (Liu et al., 2012). Thus, decreased expression of the *LRRC26* gene is associated with an unfavorable prognosis in PC, which is consistent with our results.

Correlation analysis of gene expression with PFS in CRPC revealed that the following genes had the strongest Spearman correlation coefficients: *CLPB* (*r_s_* = −0.49), *PRPF19* (*r_s_* = −0.43), *MPST* (*r_s_* = 0.41), *IFNGR1* (*r_s_* = 0.44), *CTAGE5* (*r_s_* = 0.42), *GIMAP6* (*r_s_* = 0.40), and *BET1* (*r_s_* = 0.40).

The caseinolytic mitochondrial matrix peptidase chaperone subunit B (*CLPB*) is an ATPase associated with a variety of cellular processes. Currently, there are no data on the role of this gene in cancer. Our study showed that an increase in expression was associated with an unfavorable prognosis, as evidenced by the strong correlation and high statistical significance.

The pre-mRNA processing factor 19 (*PRPF19*) gene encodes the hPrp19 protein, which is involved in many physiological processes, such as the ubiquitin-proteasome system, DNA damage response, proliferation, and apoptosis (Yin et al., 2012). The hPrp19 protein has been reported to play a potential pro-oncogenic role due to its proliferation-promoting activity. hPrp19 is also required for the expression of p21, which has an intense cell cycle arrest-promoting effect (Chen et al., 2011). The mechanism of hPrp19 in cancer is still unclear; however, its involvement in DNA repair is expected to be correlated with tumor progression. According to our results, in CRPC, increased expression of the *PRPF19* gene is associated with early relapse.

The mercaptopyruvate sulfurtransferase (*MPST*) gene encodes an enzyme involved in the catalysis of endogenous hydrogen sulfide from L-cysteine. Various studies have shown that endogenous hydrogen sulfide can regulate the occurrence and development of tumors and can participate in cancer progression by stimulating angiogenesis and cell growth in colon and ovarian cancer (Bhattacharyya et al., 2013; Szabo et al., 2013; Hellmich and Szabo, 2015). In CRPC, we observed an increase in *MPST* expression at relapse, which suggests the involvement of this gene in PC progression.

Interferon-gamma receptor 1 (*IFNGR1*) encodes a subunit of the IFN-γ receptor that acts in IFN-γ pathways and regulates the immune response. Reduced expression of this gene was observed in MYC-dependent metastatic PC. Experiments that activate *IFNGR1* gene expression demonstrated strong activation of tumor-suppression signaling and sustained apoptosis (Wee et al., 2014). We observed similar results in CRPC, which showed decreased expression of the *IFNGR1* gene in tumor samples with early relapse.

The CTAGE family member 5 (*CTAGE5*) gene product (an ER export factor) is involved in the transport of collagen VII in the endoplasmic reticulum. In PC, the *CTAGE5* gene is involved in tumor-specific splicing (Ren et al., 2012). Our study showed a decrease in the expression of *CTAGE5* in samples with early relapse.

The GTPase, IMAP family member 6 (*GIMAP6*) gene encodes a protein belonging to the GIMAP family of proteins, which are mainly expressed in immune system cells and contribute to the development of thymocytes, apoptosis of peripheral lymphocytes, and T-helper differentiation (Filen et al., 2009). Dysregulation of *GIMAP6* expression is observed in non-small cell lung cancer, where the *GIMAP6* gene expression is decreased in tumor samples (Shiao et al., 2008). Decreased expression of the *GIMAP6* gene has also been noted in hepatocellular carcinoma (Huang et al., 2016). In CRPC, we also observed that decreased expression of *GIMAP6* was associated with the onset of disease relapse.

Bet1 Golgi vesicular membrane trafficking protein (*BET1*) is a membrane protein associated with the Golgi complex that is involved in vesicular transport. There are few published studies on the function of this gene in cancer. There is a report that *BET1* was identified as part of a gene expression signature associated with a favorable prognosis in glioblastoma (Cai et al., 2020). We showed that decreased *BET1* expression was associated with early relapse in CRPC. In addition to the identified protein-coding genes, in our correlation analysis, we also found long non-coding RNAs (lncRNAs) with altered expression patterns. LncRNAs are involved in the regulation of various biological processes, including many cancer-associated pathways (Spizzo et al., 2012; Flynn and Chang, 2014; Quinn and Chang, 2016). Aberrant lncRNAs expression has been observed in a variety of cancers, and many studies have shown a link between these molecules and cancer development and progression. In LAPC, we found changed expression of the following lncRNAs that were associated with PFS: *MIR22HG* (*r_s_* = 0.27), *MEG3* (*r_s_* = 0.27), and *MIR29B2CHG* (*r_s_* = 0.31).

Regarding the role of MIR22 host gene (*MIR22HG*) in PC, decreased *MIR22HG* expression has been shown to be significantly associated with a higher Gleason score and shorter PFS time, highlighting its prognostic potential (Shen et al., 2019). In our analysis, we also observed an association between decreased *MIR22HG* expression and early onset of disease recurrence.

Studies on maternally expressed 3 (*MEG3*) have shown that this lncRNA inhibits the proliferation and metastasis of gastric cancer through p53 signaling (Wei and Wang, 2017). In PC, *MEG3* is characterized by a downregulated expression, and increased expression has an inhibitory effect on tumor growth (Wu et al., 2019). Therefore, our results are consistent with the published data.

There are currently no data on the role of MIR29B2 and MIR29C host gene (*MIR29B2CHG*) in cancer. Therefore, we report here, for the first time, the involvement of *MIR29B2CHG* in the progression of LAPC.

In CRPC, we identified an association between an increase in the expression of *LINC02001* (small nucleolar RNA host gene 30 [*SNHG30*]; *r_s_* = −0.38) and early onset of disease relapse. No studies on the involvement of *LINC02001* in cancer have been reported.

Summing up, in this study, genes and pathways associated with PFS in advanced-stage PC (LAPC and CRPC) were identified. Our results are consistent with the previous studies that reported the participation of *KIF11*, *SQLE*, *SOD3*, *LRRC26*, *IFNGR1*, *MIR22H6*, and *MEG3* in the carcinogenesis and progression of PC. The possible association with the progression of PC was first shown for genes *ZC2HC1A*, *CLPB*, *PRPF19*, *MPST*, *GIMAP6*, *BET1*, *MIR29B2CH6*, and *LINC02001*. All listed genes showed strong correlations with PFS and thus could be considered as potential prognostic markers.

## Data Availability Statement

The study included RNA-Seq data for LAPC samples from the PRAD project (*n* = 55) and CRPC samples from the WCDT-MCRPC project (*n* = 84), which were donated to the TCGA consortium.

## Author Contributions

EAP, AVK, KMN, ADK, BYA and GTS conceived and designed the work. GSK, AAK, and VSP carried out bioinformatics analysis. MVS, MSF, and DYT carried out statistical analysis. EAP, AVS, and GSK wrote the manuscript. All authors contributed to the article and approved the submitted version.

### Conflict of Interest

The reviewer AT declared a shared affiliation with two of the authors, GS and DT, to the handling editor at time of review.

The remaining authors declare that the research was conducted in the absence of any commercial or financial relationships that could be construed as a potential conflict of interest.

## Footnotes

^1^https://portal.gdc.cancer.gov/

^2^https://www.r-project.org/

## Supplementary Material

SUPPLEMENTARY TABLE S1 List statistically significant genes according to quasi-likelihood F-test for the LAPC.

SUPPLEMENTARY TABLE S2 List statistically significant genes according to quasi-likelihood F-test for the CRPC.
